# Doctors and Dentists with American Diplomas

**Published:** 1890-05

**Authors:** 


### Doctors and Dentists with American Diplomas.

According to Dalziel's Berlin agency (says the *Pall Mall Gazette*) by direction of the Privy Council, a census is being secretly taken of the number of doctors possessing American college degrees practising medicine and dentistry in the empire. It is the intention of the Government to interdict the carrying of an American doctor's degree, a title assumed here principally by dentists. In German colleges there is no such degree as 'Doctor of Dentistry,' consequently many German students matriculate at an American university, generally in Philadelphia, Baltimore, or New York. They graduate with the degree of D.D.S. (Doctor Dental Surgery), and returning to Germany place the prefix 'Dr.' on their door plates. This is no longer to be permitted, as it is regarded as misleading to patients; an American medical degree being considered as next to valueless in Germany. In Berlin at present there are twenty-six German dentists with American diplomas. Their licenses will be taken from them unless they call themselves plain 'Mr.' If one may judge from recent expressions of opinion from the leading London dentists and from the action of the Odontological Society which systematically ignores the American degrees, we should say that there is as strong a feeling in London against the assumption by dentists of the title of Dr. as in Berlin.

